# Liquid Moisture Transport in Single and Layered Cotton Woven Fabrics

**DOI:** 10.3390/ma18102326

**Published:** 2025-05-16

**Authors:** Małgorzata Matusiak, Joanna Szpunar

**Affiliations:** Lodz University of Technology, Faculty of Material Technologies and Textile Design, Institute of Architecture of Textiles, 90-924 Lodz, Poland; szpunar999@gmail.com

**Keywords:** woven fabrics, multilayer sets, comfort, liquid transport, wetting, absorption, spreading

## Abstract

The transport of liquid sweat mainly concerns the material that is next to the skin during clothing usage. When the inner surface of the material closest to the skin is adjacent to another layer of clothing, the matter is not so clear-cut. In the case of the multilayer clothing, the outer layer receives the liquid sweat from the next-to-skin layer and transport it further to ambient air. The aim of this work was to analyze the transport of liquid moisture in multilayer sets. Measurements were performed using a Moisture Management Tester. The studies confirmed that as a result of adding another layer in the outward direction, the characteristics of the liquid moisture transport of the internal surface of the inner layer of a multilayer package change significantly. The novelty of the presented research is the demonstration that the transport of liquid moisture in the layer adjacent to human skin may change when the next-to-skin layer is not adjacent to ambient air but to textile material forming the next layer of clothing. The presented studies also proved that the transport of liquid moisture on the inner surface of the layer adjacent to the skin changes to different extents depending on the type of material used as the next outer layer of clothing.

## 1. Introduction

The basic condition that clothing should meet during use is guaranteed user comfort. The concept of comfort has never been quantitatively defined because it depends on many variables. Each of these features is subjective, assessed based on the individual feelings of clothing users. In a broader context, it can be defined as a subjective sense of satisfaction with wearing clothing, resulting from clothing meeting a set of requirements during its use. The exact definition of comfort is complicated because it is a matter of individual preferences and organoleptic sensations. In identical conditions, one person may feel completely comfortable while another may not feel this comfort at all. The comfort of clothing usage can be divided into different types, satisfying the needs of the user at various levels [1]. The following four types of clothing comfort can be distinguished [2]:

- Physiological;
- Psychological;
- Sensory;
- Fitting.

Among the types of comfort listed above, physiological comfort plays a key role. It applies to all types of clothing, regardless of the purpose and conditions of usage.

Physiological comfort, also known as thermo-physiological comfort, is the most important criterion for assessing clothing products that are designed to ensure the optimal functioning of the human body depending on the conditions of usage [3]. Thermo-physiological comfort is a state of satisfaction with the thermal conditions of the environment, where full freedom of movement and mental agility are ensured. This concept also includes the creation of optimal microclimatic conditions in the layer close to human skin, which translates into the user feeling in harmony with the surrounding environment.

Various factors directly affect the thermo-physiological comfort of clothing during use. These factors can be divided into three groups according to their origin [4]:

- Factors related to the human body;
- Factors related to the environment;
- Factors related to clothing.

From the perspective of clothing design and production, clothing-related factors are the most critical. These include the comfort-related properties of textile materials used in clothing, such as the following [5]:

- Air permeability;
- Thermal resistance;
- Moisture transport.

Moisture transport is related to the removal of sweat secreted by the human body [6]. This issue should be analyzed in two aspects: the transport of water vapor and the transport of liquid moisture [7,8,9]. Liquid sweat occurs when sweat released in the form of vapor is not completely evaporated but partially condenses on the human skin. This situation occurs during intense physical exertion, for example, when performing heavy work or while engaged in sports. Stress is also a factor that causes intense sweating. The water vapor permeability of clothing materials is the factor that is responsible for the evaporation of sweat. However, in many cases, the water vapor permeability of the material or set of materials of which an item of clothing is made is insufficient for the complete evaporation of sweat, in which case the sweat condensed on the skin must be absorbed by the clothing material and carried away to the outside. The ability of textile materials to transport liquid sweat is responsible for this.

Studies on the transport of liquid moisture in textile materials have been the subject of numerous works published to date [6,7,8,9,10,11,12,13,14,15,16,17,18,19,20,21,22,23]. In the published studies, most attention has been paid to single-textile materials, especially knitted fabrics, which are used in underwear or sportswear [14,15,16,17]. Öner et al. [14] performed investigations aimed at an assessment of the influence of raw materials, namely, the effect of the kind of weave and tightness of structures on the absorption of liquid by knitted fabrics of different weaves made of cotton, viscose and polyester. They found that polyester fabrics have a higher OMMC (Overall Moisture Management Capacity) than knitted fabrics made of cellulose fibers. Sai Sangurai et al. [15] found that the cross-section of polyester filaments significantly affects the ability of fabrics to transport liquids. Matusiak and Sukhbat [16] performed investigations of liquid moisture transport in knitted fabrics in unstretched and stretched form. They stated that stretching knitted fabrics changes their surface characteristics and the tightness of the structure, which influences moisture transport. There are also publications concerning single woven fabrics and the possibility of their transporting liquid moisture [7,20,21,22,23]. These studies are concerned mostly with the influence of woven fabric structures—weave and thread density—on moisture transport characteristics. Magnat et al. [20] found that the type of weft yarns and washing processes affect moisture transport in denim fabrics. Çeven et al. [21] studied fabrics made of linen and linen–polyester yarns (80% polyester and 20% linen) with different weft counts. They found that the structural properties of fabrics, such as the type of weft yarn, weft yarn count, weft yarn linear density, as well as the types of fibers and their percentages, influenced the moisture transport capacity of the tested fabrics. Matusiak and Kamińska [7] confirmed that the weave and weft linear density significantly influence the parameters characterizing the liquid moisture transport in cotton woven fabrics. Matusiak [22,23] described in detail the liquid moisture transport performance of cotton seersucker woven fabrics with varying structures. Most published research works focused on individual fabrics, particularly those specifically designed for garments worn next to the skin. Researchers have emphasized that the transport of liquid sweat primarily involves clothing materials in direct contact with the wearer’s skin during use.

This is justified by the fact that the layer of clothing adjacent to the wearer’s skin is in direct contact with the liquid sweat condensed on the skin. When evaluating textile materials in terms of the transport of liquid sweat, it is essential to consider the phenomena occurring on both surfaces of the fabric: the inner surface which lies directly against the skin and the outer surface. The efficient and rapid transport of liquid sweat from the inner to the outer surface of the material combined with a large moisture-spreading area on the outer surface facilitates the evaporation of liquid sweat into the surrounding environment. This assumption holds true when the outer surface of the material is exposed to ambient air. However, the situation becomes more complex when the outer surface of the next-to-skin layer is in contact with another layer of clothing rather than open air. In multilayer clothing systems or complete clothing ensembles, subsequent layers must receive and transport liquid sweat from the inner layer outward toward the ambient environment. Matusiak and Sukhbat [9] highlighted this phenomenon in their study of multilayer protective clothing assemblies for firefighters, which included underwear. Their research demonstrated that the transport of liquid moisture through the knitted fabrics used as underwear varied depending on the materials used in the outer layers of the multilayer system.

In the transport of liquid moisture through multilayer textile systems, the adhesion of the materials forming each layer plays a crucial role. When the individual layers of a multilayer clothing system do not adhere to one another, the outer layers cannot effectively receive liquid sweat from those closer to the wearer’s skin. However, such cases are extremely rare. Textile materials are flexible, easily deformable, and drapable. When worn, clothing hangs on the wearer’s body, resting on so-called support areas. Due to their own weight and the wearer’s movement, the layers of the multilayer system generally remain in contact with each other for most of the time the clothing is worn. As a result, the outer layers influence the transport of liquid sweat, not only in the innermost layer but also trough the successive layers, all the way to the garment’s outer surface exposed to ambient air.

Taking the above into account, the phenomenon of liquid moisture transport in multilayer clothing systems should not be overlooked when analyzing the moisture transport in clothing materials. Examining the behavior of materials intended for the layer closest to the body, in conjunction with those used in subsequent layers, can yield valuable information necessary for the effective design and production of clothing that ensures thermo-physiological comfort.

The aim of this study was to analyze the transport of liquid moisture in multilayer fabric systems. Tests were conducted on individual fabrics as well as on systems composed of two and three layers of the same fabric. Additionally, liquid moisture transport was examined in a two-layer system composed of fabrics with differing liquid moisture transport characteristics.

The novelty of this research lies in demonstrating that the liquid moisture transport in the layer directly in contact with the human skin can change when this layer is not exposed to open air but is instead covered by another textile layer. The study also showed that the extent of this change depends on the type of material used as the outer layer. This phenomenon has not been previously analyzed, and some researchers have even asserted that only skin-adjacent materials should be tested for liquid moisture transport. The findings of these studies challenge such assumptions and provide a foundation for further research on liquid moisture transport in multilayer clothing systems.

## 2. Materials and Methods

**Figure 1:**
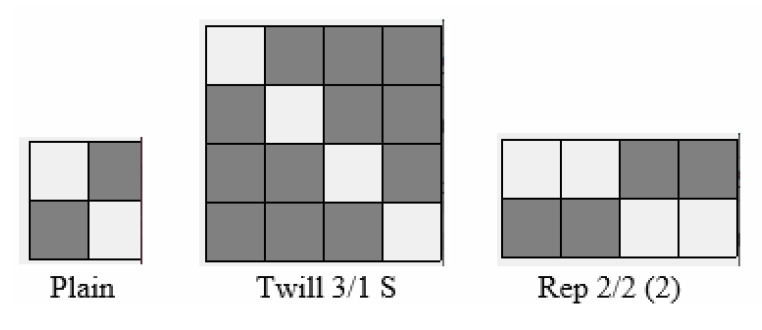
Repeats of weaves applied in the investigated woven fabrics.

**Figure 2:**
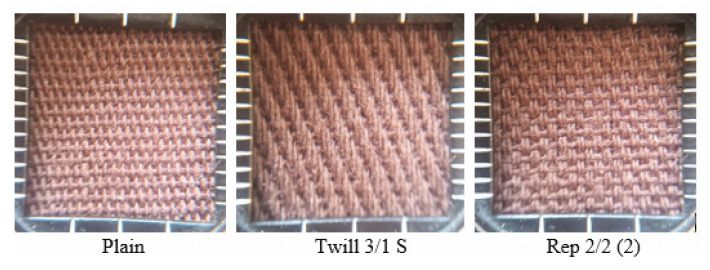
Pictures of the investigated fabrics in the weaver’s magnifying glass.

The conducted research analyzed the transport of liquid moisture through three variants of woven fabrics made of cotton, differing in terms of weave. The fabrics were manufactured on the base of the same warp—50 tex CO OE (Open End) and the same weft—60 tex CO OE yarns. The applied weaves are the following: plain, twill 3/1 S and rep 2/2 (2). Figure 1 presents the repeats of the applied weaves, whereas Figure 2 shows pictures of the investigated woven fabrics in the weaver’s magnifying glass.

All fabrics were finished in the same way. Table 1 below provides a detailed overview of the tested fabrics along with their basic parameters. The measurements of the basic structural parameters of the tested fabrics were performed in accordance with the procedures described in the relevant standards:

- Measurement of mass per unite area—PN-ISO 3801:1993 [24];
- Measurement of warp, weft density—PN-EN 1049-2:2000 [25];
- Measurement of fabric thickness—PN-EN ISO 5084:1999 [26].

**Figure 3:**
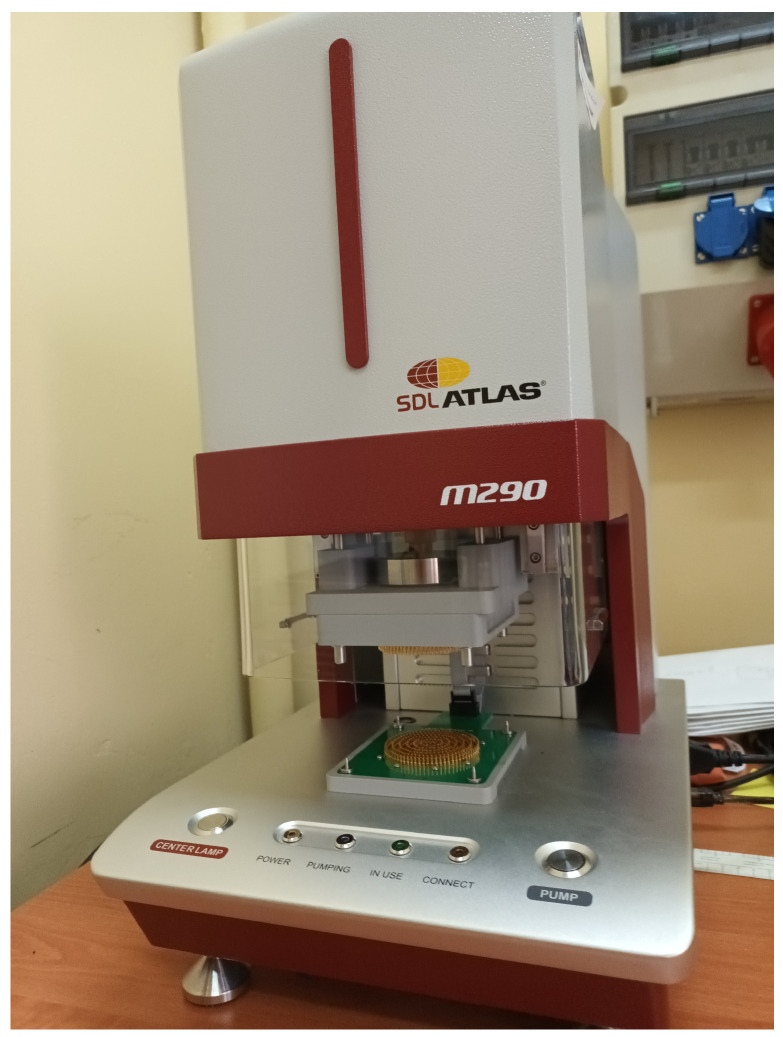
The Moisture Management Tester m290.

Measurement of parameters characterizing the liquid moisture transport in fabrics was performed by means of the Moisture Management Tester (MMT) model m290 (Figure 3) manufactured by the SDL Atlas (Rock Hill, SC, USA) [7,9,27,28,29]. Measurement was conducted according to the procedure described in the AATCC Test Method 195-2011 standard [28].

**Figure 4:**
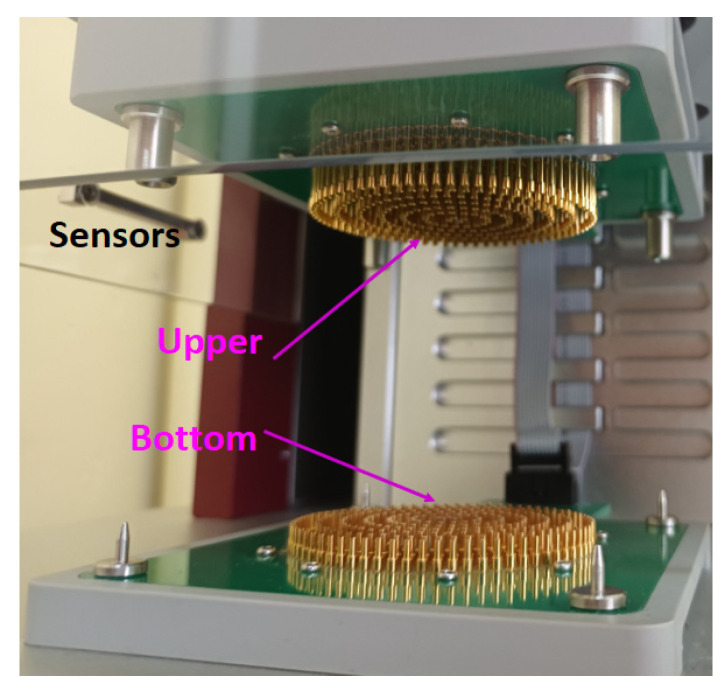
The sensor of the Moisture Management Tester m290.

The samples tested using the MMT device have a standardized size of 80 mm × 80 mm. Five measurements are performed for each of the tested variants [27,28]. The specimen for measurement is placed horizontally in the device between the upper and lower sensors (Figure 4).

The entire measurement lasts 120 s. During the first 20 s, a solution of synthetic sweat is applied to the upper surface of the sample. The solution is dropped onto the center of the upper side of the sample, which represents the surface in contact with the user’s body. The applied testing solution is transported through the sample in 3 directions:

- Spreading of moisture on the upper surface of the fabric;
- Transport of moisture through the fabric from the upper surface to the lower surface;
- Spreading of moisture on the lower surface of the fabric.

The measurements were conducted under normal climatic conditions usually applied in measurement of textile materials, namely a temperature of 20 ± 2 °C and a relative humidity of 65 ± 5%.

The device is used in the textile industry to assess the properties of textile materials in terms of their ability to transport liquid moisture. This enables measurements in the following aspects:

- Moisture absorption rate;
- Ability for unidirectional moisture transport;
- Rate of spreading liquid moisture [27].

For each of the above aspects, both the outer and inner surfaces of the fabric are tested, assuming that the inner surface is considered as the one located on the body side.

The device is computer-controlled using specialized MMT. Software version 5.1.5 installed. This software, based on the measurement of changes in fabric moisture, which cause changes in the amount of current flowing, calculates the ability of fabrics to transport liquid moisture. Then, it classifies the tested materials into one of seven classes:

- Water proof;
- Water repellent;
- Slow absorbing and slow drying;
- Fast absorbing and slow drying;
- Fast absorbing and quick drying;
- Water penetration;
- Moisture management [27].

Using the MMT device, the following parameters can be measured [27]:

- WTT—Wetting Time of Top surface, in s,
- WTB—Wetting Time of Bottom surface, in s,
- TAR—Absorption Rate of Top surface, in %/s,
- BAR—Absorption Rate of Bottom surface, in %/s,
- SST—Spreading Speed of Top surface, in mm/s,
- SSB—Spreading Speed of Bottom surface, in mm/s,
- MWRT—Max Wetted Radius on Top surface, in mm,
- MWRB—Max Wetted Radius on Bottom surface, in mm,
- R—Accumulative One-Way Transport Index, in %,
- OMMC—Overall Moisture Management Capacity (unitless).

**Figure 5:**
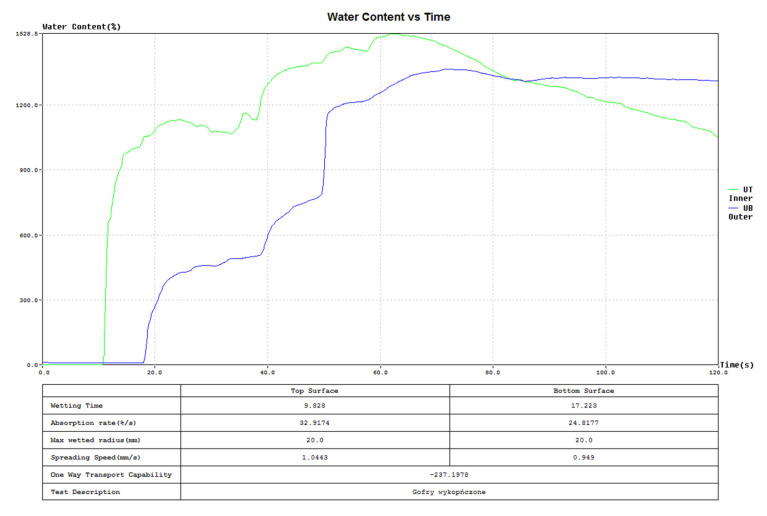
The exemplary graph “Water Content vs. Time” from the MMT: green line—water content vs. time on the top surface, blue line—water content vs. time on the bottom surface.

The first eight parameters describe the transport of liquid moisture on both surfaces of the tested sample: the upper and lower. They are measured directly by analyzing changes in the sample’s electrical conductivity resulting from variations in moisture content. The last two parameters characterize the overall performance of the entire sample. These are not measured directly but are calculated. The R parameter is derived from the curve “Water content vs. time” (Figure 5) for both the upper and lower surfaces of the sample.

The R parameter, accumulative one-way transport index, is calculated according to the equation:

(1) $$R=\frac{Area\;under\;bottom/blue\;curve-Rea\;under\;top/green\;curve}{Total\;testing\;time}$$

The OMMC parameter is calculated on the basis of three other parameters, BAR, SSB and R, according to the algorithm developed by the manufacturer of the MMT device [27,28]. In the algorithm, the BAR and SSB parameters have a weight of 0.25, and the parameter R has a weight of 0.5 based on the following equation:

(2) $$OMMC=0.25\cdot {BAR}_{ndv}+0.5\cdot R_{ndv}+0.25\cdot {SSB}_{ndv}$$

where BAR_ndv_, R_ndv_ and SSB_ndv_ are calculated as non-dimensional values using equations based on their relationships to observed maximum and minimum values. When a parameter equals the maximum observed value, it is assigned a value of 1; when it equals the minimum, it is assigned a value of 0. For parameter values between the minimum and maximum, the non-dimensional value (X_ndv_) is calculated using the following formula:

(3) $$X_{ndv}=\frac{X-X_{min}}{X_{max}-X_{min}}$$

where: X—one of the parameters: BAR, SSB, R.

Thus, the value of the OMMC parameter ranges from 0 to 1. A higher OMMC value indicates better fabric performance in liquid moisture transport.

The MMT software cooperating with the tester calculates the values of the R and OMMC parameters using built-in algorithms.

The spreading speed (SS) is calculated using the following equation [27,28,29]:

(4) $$SS=\sum_{i=1}^{N}\frac{R}{t_{i}-t_{i-1}}$$

where:

R—sensor ring radius;

N—maximum wetted rings;

t_i_—time in which the ring I is wetted.

The AATCC Test Method 195-2011 standard [28] outlines several limitations of the MMT method, including:

- The test method focuses on liquid moisture transport in the fabric’s flat state;
- It does not measure gaseous moisture transport properties (e.g., water vapor transmission);
- The method may not be applicable to coated, laminated, or complex fabric constructions.

Additionally, it is very difficult to measure fabrics with a loose structure and high porosity. In such a case, the test fluid may pass through the pores in the material via gravity, collecting on the surface of the lower sensor without being absorbed. As a result, it becomes impossible to determine the amount of fluid that was not absorbed by the material. This can cause significant measurement errors [29].

All the above parameters of fabrics and their sets were determined using the MMT. Among these, the parameters that best reflect the effect of additional layers on liquid moisture transport at the inner surface, i.e., the side adjacent to the skin surface of the first fabric, were selected for discussion. The following parameters were discussed: WTT, TAR, SST, SSB, MWRT, MWRB and OMMC. Two parameters, WTB and BAR, were not included in the discussion. However, the values of all measured parameters are presented in the Supplementary Materials: Tables S1–S10.

For each fabric variant, the measurements were conducted on single-layer fabric as well as on two- and three-layer assemblies. Additionally, measurements were performed on a two-layer assembly composed of fabrics No. 1 (plain) and No. 2 (twill). In this part of the experiment, the two-layer set was created from samples No. 1 and No. 2, as presented in Table 1.

**Figure 6:**
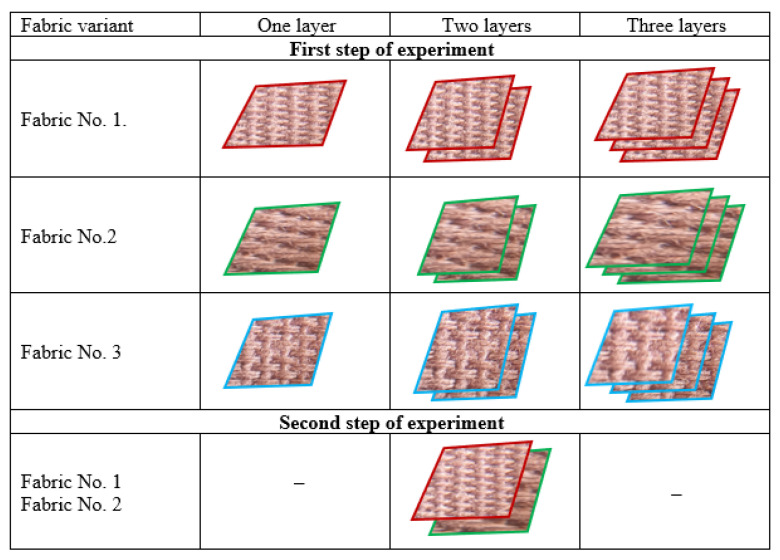
The arrangement of the performed investigations.

The arrangement of the investigations is presented in Figure 6. In all stages of the investigations, the samples were placed in such a way that the right side of the fabric was facing up.

The results were analyzed using the statistical tools—multifactor analysis of variance (ANOVA).

## 3. Results and Discussion

A total of 10 variants of material were tested: 3 individual fabric variants, 4 variants of two-layer sets and 3 variants of three-layer sets (Figure 4). For each variant, five repetitions of measurement were performed. From each measurement, the values of ten parameters were received, resulting in a large dataset. Detailed results are presented in the Supplementary Materials: Tables S1–S10.

**Figure 7:**
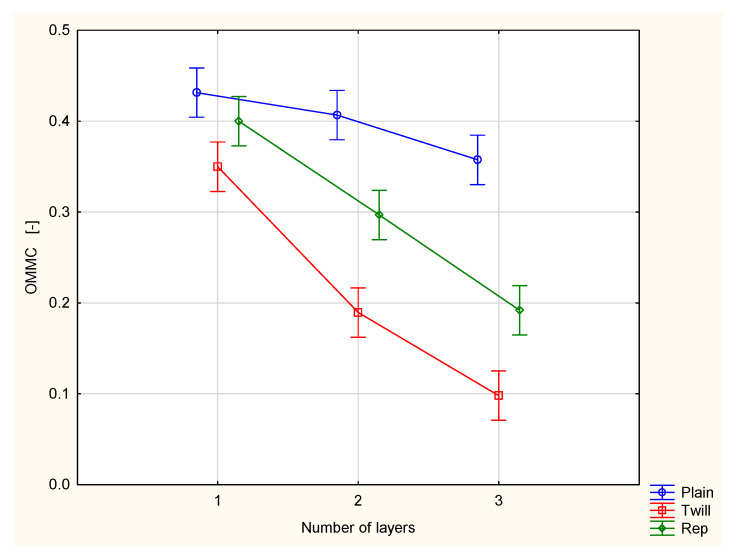
Overall Moisture Management Capacity of the investigated woven fabrics and their two- and three-layer sets.

Figure 7 presents the Overall Moisture Management Capacity (OMMC) of the investigated woven fabrics and their two- and three-layer sets.

The Overall Moisture Management Capacity (OMMC) is a calculative parameter. It is calculated based on the absorption rate of bottom surface (BAR), accumulative one-way transport index (R) and spreading speed of bottom surface (SSB) according to the formula defined by the MMT manufacturer [27]. The OMMC parameter indicates the overall ability of fabric being investigated to transport liquid moisture. The value of the OMMC parameter ranges from 0 to 1, where a higher OMMC indicates a better capacity for liquid moisture transport. On the basis of the obtained results, it was stated that the fabrics being investigated are different from each other in the range of the OMMC value. The highest value was obtained for the plain woven fabric (OMMC = 0.43), the lowest was recorded for the twill fabric (OMMC = 0.35). Additionally, the OMMC value decreases as the number of layers increases (Figure 7). For each weave type, the OMMC value of the three-layer set is significantly lower than that of the two-layer set. This is expected, as more layers mean a larger/thicker barrier for liquid moisture being transported from the inner (top) surface of the set to the outer (bottom) surface.

It should be noted, however, that the greatest decline in moisture transport ability occurred with the twill weave fabric, where the OMMC value dropped from 0.35 for the single layer, to 0.19 for the two-layer system, and further to 0.10 for the three-layer system. In contrast, the plain weave fabric showed the smallest decrease, with OMMC values declining from 0.45 to 0.41 and then to 0.36. The rep weave fabric exhibits decreases in the OMMC that were smaller than those of twill weave but greater than those of the plain weave (Figure 7).

On the basis of the OMMC value, it can be stated that the value of the OMMC parameter depends on the fabric weave. Different weaves vary in the arrangement and interlacing pattern of warp and weft threads. The basic unit of a weave structure is the repeat, and within each repeat, the number of warp and weft threads passing from one side of the fabric to the other differs depending on the weave type. This is probably the decisive factor, which makes fabrics of different weaves transport moisture in different ways. In the context of liquid sweat transport, the goal is to move the sweat condensed on human skin to the outer surface of the clothing, where it can evaporate into the surrounding air. The greater the number of yarn transitions in a unit of length in both directions of the fabric (or in a unit of fabric surface), the better the transport of sweat from the inner surface (close to the skin) to the outer surface. Therefore, when assessing a fabric’s ability for liquid moisture transport, it is more meaningful to consider the number of interlacing points per unit of length or surface area, rather than simply comparing weave repeats. This is because weave repeats can vary in size and thread count, making direct comparisons unreliable unless normalized by fabric dimensions.

In the case of the tested fabrics, over the length corresponding to 4 warp threads and 4 weft threads in each direction, the following was observed:

- In the plain weave—4 warp passes and 4 weft passes;
- In the twill 3/1 weave—2 warp passes and 2 weft passes;
- In the rep 2/2 (2) weave—4 warp passes and 2 weft passes.

Among the fabric variants investigated, the plain woven fabric shows the best performance in terms of liquid moisture transport. The value of the OMMC parameter was the highest for the single-layer plain fabric as well as its two- and three-layer sets. This is due to the fact that the plain weave fabric, compared to twill and rep weave fabrics of the same warp and weft density, has the greatest number of yarn passes (warp and weft) from one side of the fabric to the other in a unit of length in both directions. Such a structure facilitates the more efficient flow of moisture from the upper (inner) to the lower (outer) surface of the fabric. In contrast, the fabric with a 3/1 S twill weave performed the worst. Among the fabrics studied, the twill weave fabric has the fewest number of interlacing point passes per unit area. The repeat of this fabric contains long floats of warp yarns (Figure 2) that run along the fabric’s surface. This results in capillaries between the fibers within the warp yarns and between yarns themselves being predominantly oriented in a horizontal plane. Consequently, liquid moisture tends to spread across the upper surface rather than being transported to the bottom (outer) surface. This behavior is undesirable, as it impedes the removal of liquid sweat from the wearer’s skin to the external environment.

Statistical analysis using two-way ANOVA confirmed that the influence of weave and number of layers influence the OMMC value in a statistically significant way. The interaction of both independent variables, i.e., weave and number of layers, is also statistically significant at the 0.05 significance level.

From the perspective of ensuring thermo-physiological comfort, the phenomena occurring on the inner (upper) surface of the fabric or fabric system are particularly important, because this surface is in direct contact with the user’s skin and shapes his or her sense of comfort.

**Figure 8:**
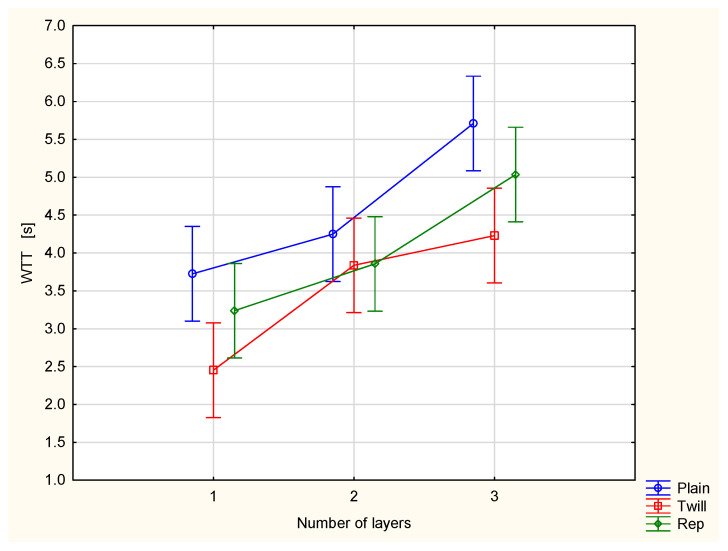
Wetting time of the top surface of the investigated woven fabrics and their two- and three-layer sets.

Figure 8 presents the wetting time of the top surface (WTT) of the woven fabrics and their multilayer sets.

The parameter presents the time at which the top surface begins to be wetted after the dosing of test liquid stars [27,28]. Wetting is the initial stage of fluid spreading across the fabric surface. The interpretation is as follows: a shorter wetting time indicates a better ability of fabric to manage liquid moisture. This is because wetting governs the transport of liquid through capillary action. The greatest wetting time was stated for the plain weave fabric and its sets. It is difficult to explain. Particular phenomena of liquid moisture transport, i.e., wetting, spreading on the surface, transport from the upper surface to the bottom surface and absorption, interact with each other. It is difficult to isolate the influence of a single factor on the observed phenomenon. In the plain weave fabric, due to the greatest number of thread interlacing points and the greatest yarn crimp, they are the most dense. This may explain the longest wetting time of the upper surface of the plain weave fabric.

Statistical analysis confirmed that both the number of layers and weave type significantly influence the value of the WTT parameter. Tukey’s test revealed that significant differences exist between the majority of compared variant pairs.

The obtained results show that adding additional layers of the same woven fabric increases the wetting time, thereby reducing the efficiency of liquid moisture transport through the fabric system. This effect is observed for each fabric variant. The wetting time of the top surface of a single plain woven fabric is 3.72 s. When an outer layer of the same fabric is added, the wetting time increases to 4.25 s. In a three-layer set of the plain woven fabric, the wetting time of the top surface, i.e., the surface next to the skin, reaches 5.71 s. These findings challenge the common assumption that only the innermost clothing layer, which is in direct contact with the human skin, influences the transport of condensed sweat. This study demonstrates that adding outer layers, even those not in direct contact with the skin, can significantly affect the moisture transport properties of the innermost layer. This conclusion is supported by additional results obtained for the top surface of the tested fabrics. 

**Figure 9:**
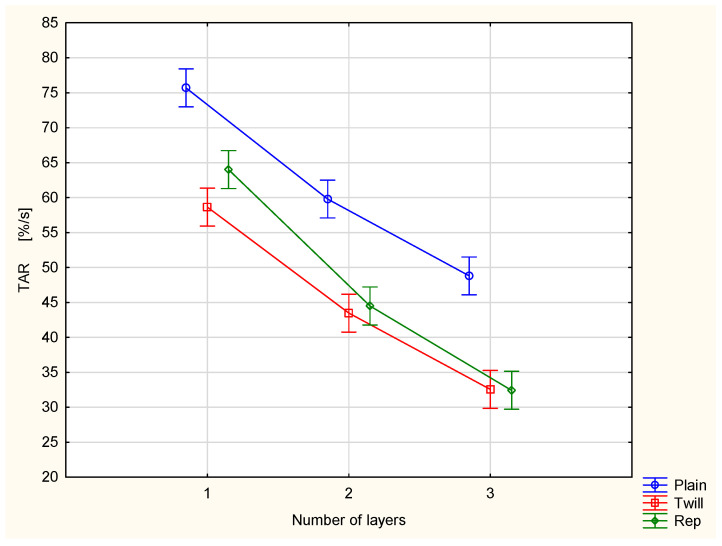
Absorption rate of the top surface of the investigated woven fabrics and their two- and three-layer sets.

Figure 9 presents the absorption rate of the top surface (TAR) for the investigated fabrics.

The TAR parameter represents an average rate of liquid moisture absorption by the top (upper) surface of the measured fabric during the initial change in water content on the surface [27,28]. The graph clearly illustrates that the absorption capacity of the top surface of the investigated woven fabrics decreases significantly with the addition of successive outer layers from the outside, i.e., from the side exposed to the surrounding air during clothing use. This phenomenon is beneficial from the perspective of physiological comfort, as it results in less liquid sweat being absorbed by the surface of the clothing in direct contact with the wearer’s skin.

**Figure 10:**
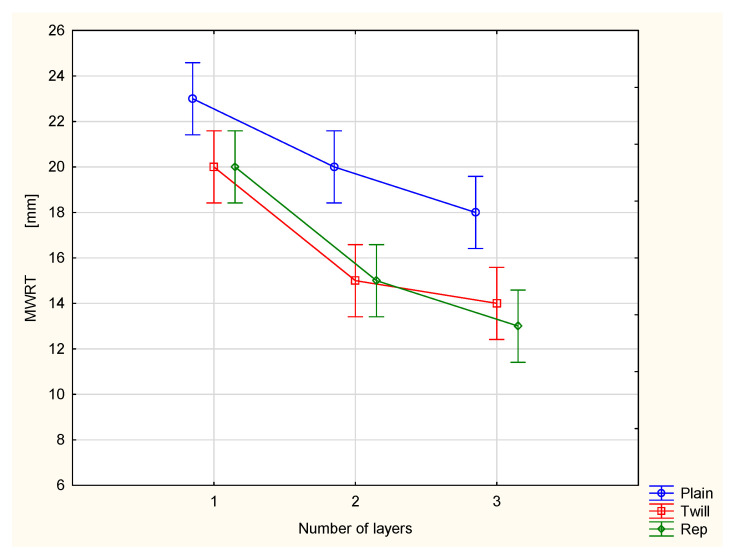
Maximum wetted radius of the top surface of the investigated woven fabrics and their two- and three-layer sets.

This is reflected in the values of the maximum wetted radius on the inner surface (MWRT) of the tested fabrics and their multilayer sets (Figure 10). The MWRT parameter refers to the maximum radius of the sensor ring on which the presence of liquid was detected. It is important to note that the trace of liquid on the fabric surface may not be perfectly circular. Its shape depends on the structure of the material being tested. A smaller wetted radius indicates a smaller area of liquid trace on the surface of the sample. On the inner surface of the material, this is beneficial from the perspective of the physiological comfort of the clothing user. A smaller wetted area means less skin contact with moisture, reducing the sensation of dampness and discomfort. Conversely, for the maximum wetted radius on the bottom (outer) surface (MWRB), a larger radius is desirable. A broader wetted area on the outer surface of the fabric facilitates faster moisture evaporation into the surrounding air, leading to quicker drying of the clothing and a more rapid reduction in the discomfort associated with wet garments.

**Figure 11:**
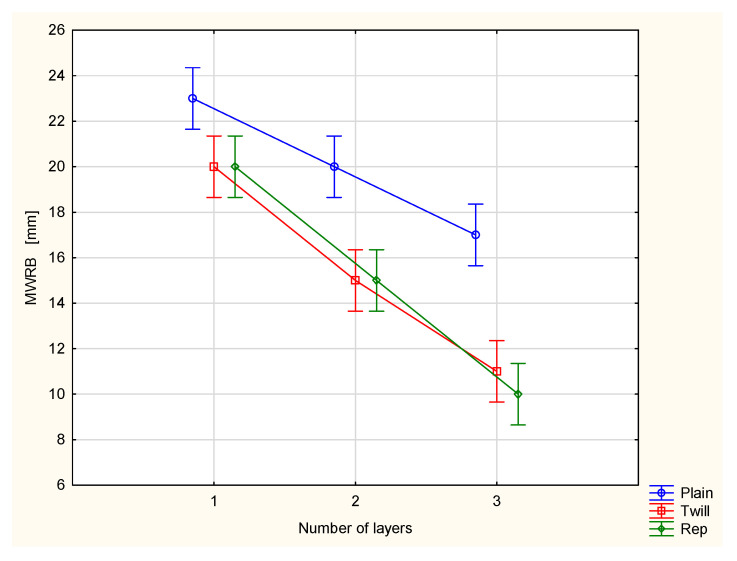
Maximum wetted radius of the bottom surface of the investigated woven fabrics and their two- and three-layer sets.

In the case of the investigated fabrics and their multilayer sets, it was stated that adding the next outer layer causes changes in both the maximum wetted radius on the top surface (Figure 10) and on the bottom surface (Figure 11). For all investigated fabrics, adding a second and third layer resulted in decreased values of both the MWRT and MWRB parameters. This has two opposing effects. On one hand, it is beneficial, as the reduced moisture trace on the inner surface, adjacent to the skin, helps minimize the sensation of discomfort. On the other hand, it also means that the evaporation of liquid sweat from the outer surface of the clothing package is slower, potentially prolonging the drying time of the clothing.

For the TAR, MWRT and MWRB cases, the differences between the variants are statistically significant at the 0.05 significance level. Statistically significant differences were also stated between the majority of pairs of compared variants.

**Figure 12:**
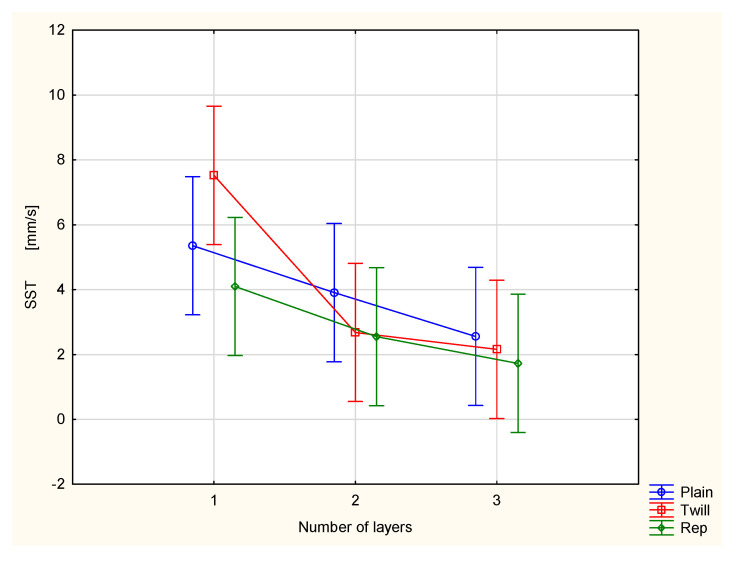
Spreading speed of the top surface of the investigated woven fabrics and their two- and three-layer sets.

**Figure 13:**
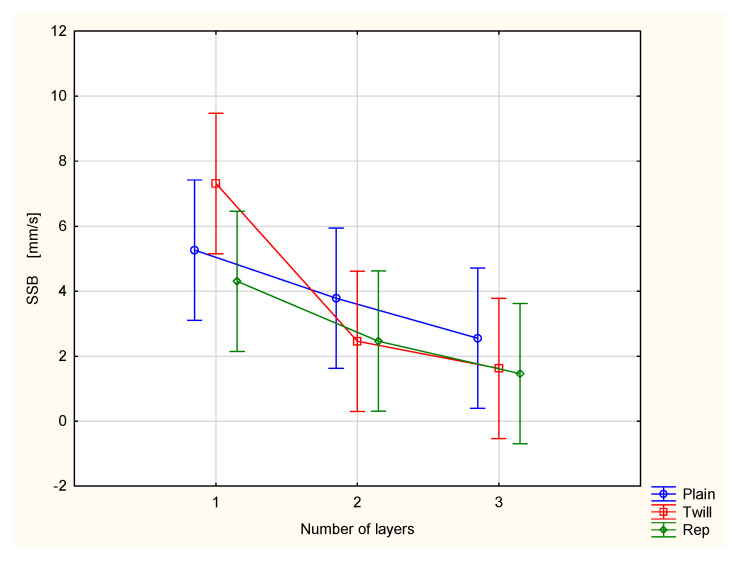
Spreading speed of the bottom surface of the investigated woven fabrics and their two- and three-layer sets.

The spreading speed on both the top surface (Figure 12) and bottom surface (Figure 13) decreases. These effects are also contradictory from the perspective of physiological comfort. Slower spreading of liquid sweat on the surface adjacent to the human skin is beneficial, as it helps maintain the moisture. However, on the outer surface, the slower spread of moisture reduces the rate of evaporation of liquid sweat into the surrounding air. This may hinder the overall cooling effect.

In both cases, i.e., the SST and SSB, a large scatter of results was observed. However, statistical analysis confirmed that the number of layers has a statistically significant effect on the values of both parameters at the 0.05 significance level. In contrast, the influence of weave on the values of the SST and SSB parameters was found to be statistically insignificant. Post hoc Tukey’s test revealed that for the SST parameter, statistically significant differences occurred only between the single-layer twill 3/1 S fabric and the remaining measured variants. For the SSB parameter, statistically significant differences were observed only between the single-layer twill 3/1 S fabric and the three-layer sets composed of twill and rep fabrics.

In next stage of the investigations, a two-layer set composed of two specimens of plain woven fabric (Fabric No. 1) was compared with a two-layer set consisting of plain woven fabric (Fabric No. 1) as an inner layer and twill 3/1 S woven fabric (Fabric No. 2) as the outer layer of the set. The aim of this part of the investigation was to assess the influence of the outer fabric variant on the liquid moisture transport behavior of the inner fabric.

**Figure 14:**
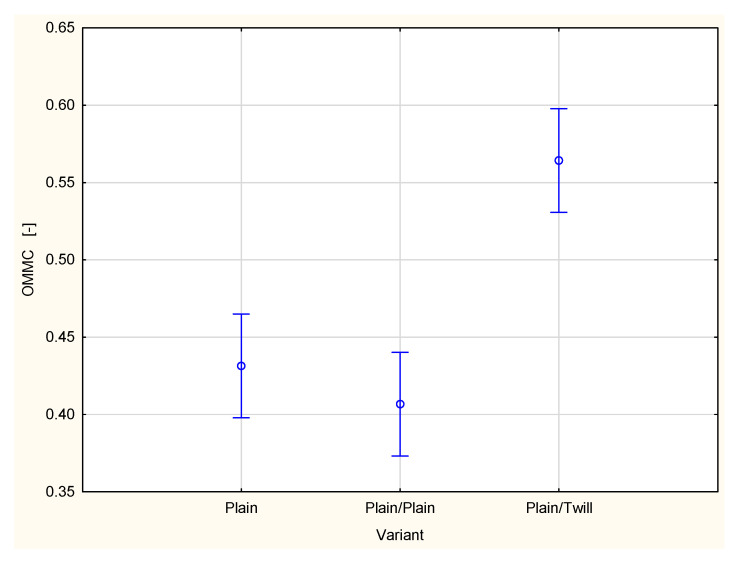
Overall Moisture Management Capacity of the plain woven fabric and its two-layer sets.

Figure 14 presents the Overall Moisture Management Capacity (OMMC) of the plain woven fabric, both on its own and in combination with a second layer, either another layer of the same fabric or a layer of twill fabric.

It is clearly evident that the variant of fabric applied as a second outer layer influences the liquid moisture transport within the fabric set. When the plain woven fabric is used as a second layer, the Overall Moisture Management Capacity of the two-layer set is lower than that of a single layer of the same plain woven fabric. In contrast, using the twill 3/1 S woven fabric as the outer layer significantly increases the value of the OMMC parameter, indicating an improvement in the liquid transport capacity of the two-layer set compared to the single layer of the plain woven fabric. Statistical analysis using one-way ANOVA confirmed that the variant of the fabric set has a statistically significant effect on the OMMC value at the 0.05 significance level. Post hoc Tukey’s test further revealed that a statistically significant difference exists between the OMMC value for the plain/twill set and those of both the single-layer plain weave fabric and the plain/plain set.

**Figure 15:**
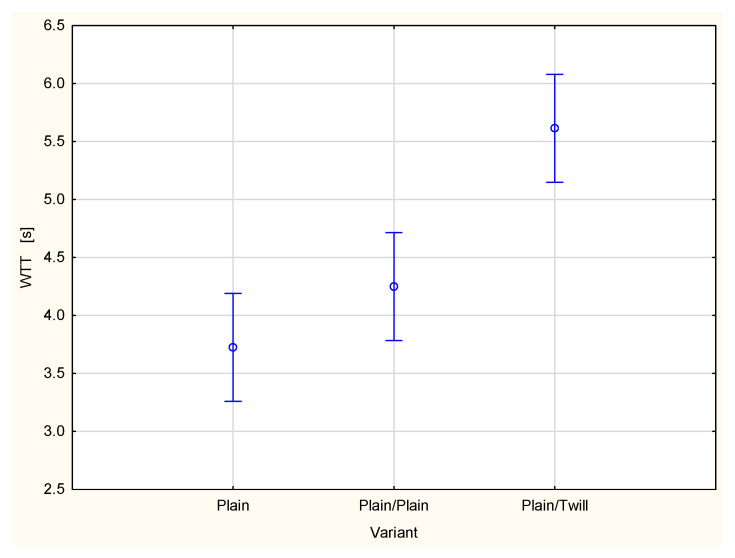
Wetting time of the top surface of the plain woven fabric and its two-layer sets.

**Figure 16:**
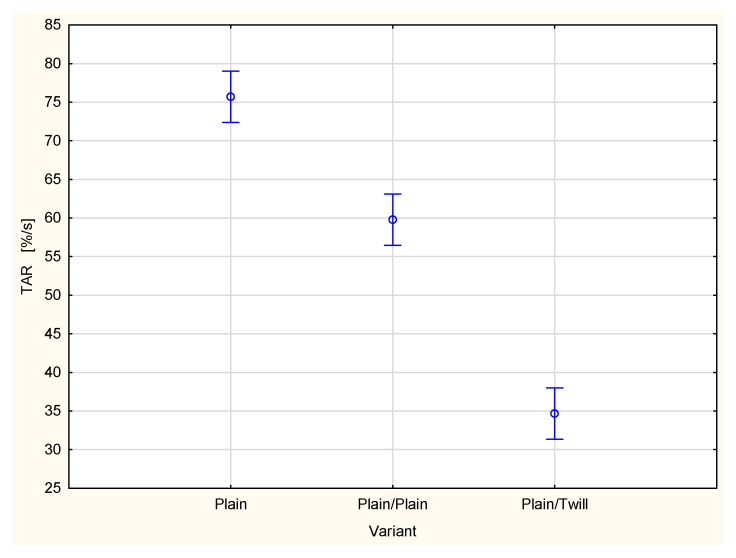
Absorption rate of the top surface of the plain woven fabric and its two-layer sets.

**Figure 17:**
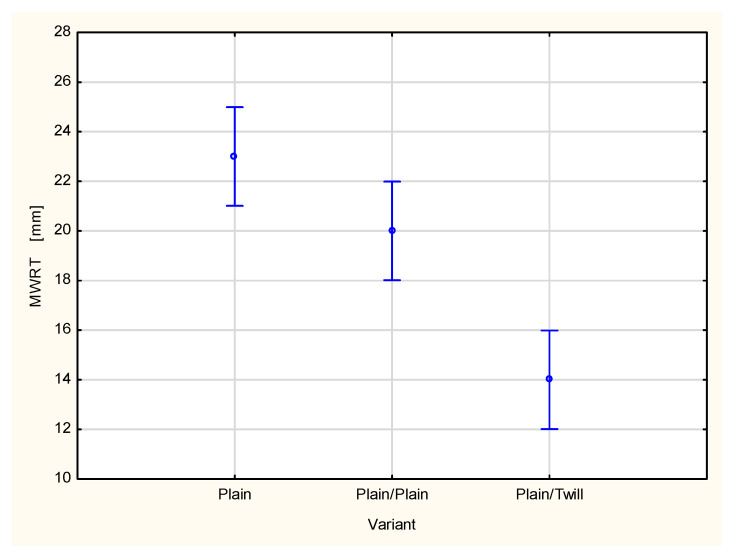
Maximum wetted radius of the top surface of the plain woven fabric and its two-layer sets.

**Figure 18:**
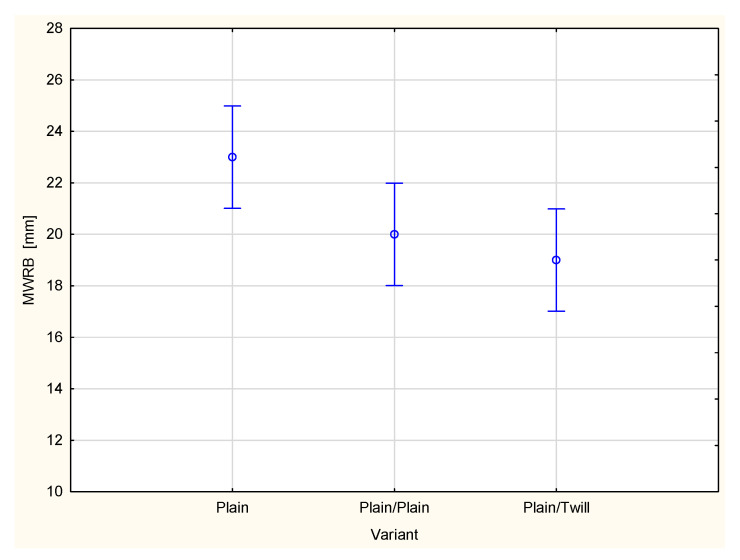
Maximum wetted radius of the bottom surface of the plain woven fabric and its two-layer sets.

**Figure 19:**
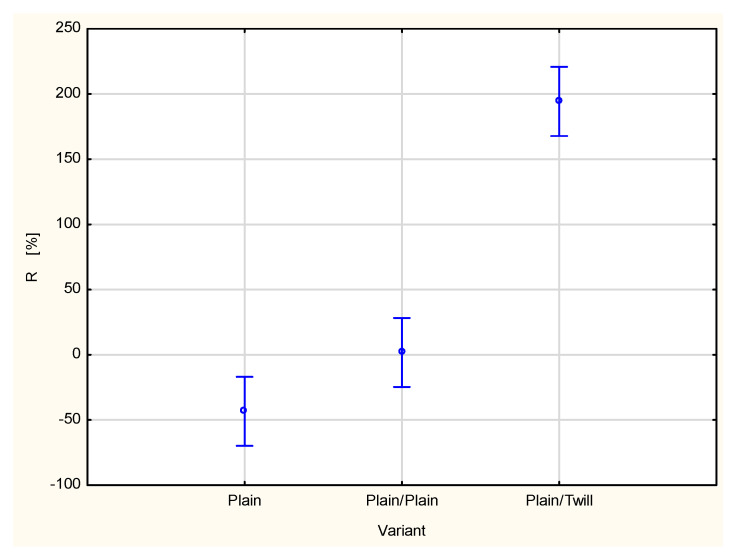
Accumulative one-way transport index of the plain woven fabric and its two-layer sets.

A type of fabric applied as a second outer layer of the two-layer set with the plain woven fabric as a first inner layer also influences other parameters related to liquid transport in the two-layer fabric set (Figure 15, Figure 16, Figure 17, Figure 18 and Figure 19). For the parameters, TAR, MWRT, MWRB and SST, the values observed for the plain/plain fabric set are significantly lower than those for the plain/twill fabric set. An exception is the wetting time of the top surface (WTT, Figure 15), which is substantially higher for the plain/twill set compared to the plain/plain set. Similar to the OMMC results, the post hoc Tukey’s test for the WTT values confirmed that a statistically significant difference exists between the plain/twill set and both the single-layer plain weave fabric and plain/plain fabric set.

Combining the plain fabric with twill 3/1 S fabric significantly reduced the moisture absorption rate of the top surface (TAR) of the inner plain fabric from 75.7%/s for the single-layer fabric to 34.9%/s for the plain/twill fabric set (Figure 16). Statistical analysis (ANOVA and Tukey’s test) confirmed that the differences between the fabric variants are statistically significant at the 0.05 significance level.

The maximum wetted radius for both the top (MWRT) and bottom (MWRB) surfaces decreased in both the plain/plain fabric set and the plain/twill set compared to the values observed for a single layer of the plain woven fabric (Figure 15 and Figure 16). For the inner surface, this is a beneficial phenomenon, as previously noted. A significantly greater decrease in the maximum wetted radius on the top surface was observed for the plain/twill set, much greater than that for the plain/plain set. On the bottom surface, the reduction in the maximum wetted radius was smaller, and the difference in the MWRB values among the fabric’s sets was less pronounced than on the top surface of the two-layer sets. This was confirmed through statistical analysis. Specifically, differences in the MWRT parameter were statistically significant across all variants, whereas for the MWRB parameter, a statistically significant difference was found only between the single plain weave fabric and the plain/twill fabric set.

An analysis of the individual parameters characterizing the transport of liquid moisture shows that combining two layers of woven fabrics generally worsened the conditions of moisture transport. However, the value of the OMMC parameter increased for the plain/twill fabric set compared to the single-layer plain fabric (Figure 7). This improvement is likely due to the value of the R parameter (Figure 19). The R parameter, or accumulative one-way transport index, represents the difference in the accumulated moisture content between the outer and inner surfaces of the fabric. A higher R value indicates more efficient liquid moisture transport from the inner (next to skin) surface to the outer surface of the measured fabric, which is beneficial for physiological comfort [14]. A high and positive R value means that the liquid content on the outer surface exceeds that on the inner surface. The value of the R parameter for the plain/twill fabric set is significantly higher than that for both the plain/plain fabric set and the single-layer plain woven fabric (Figure 18). This explains the corresponding increase in the OMMC values, as the R parameter is a kay component in the OMMC calculation algorithm, carrying the highest weights (0.5) and, thus, having the greatest influence on the OMMC value.

Statistical analysis by means of the one-way ANOVA confirmed that the influence of the sample variant on the R value is statistically significant at the 0.05 significance level. The post hoc Tukey’s test confirmed further that statistically significant differences occur between the R values for both the plain/twill variant and the single plain woven fabric and plain/plain variant. The difference between the R value for the single-layer plain woven fabric and plain/plain set is statistically insignificant.

## 4. Conclusions

The obtained results challenge the commonly held belief that, in liquid moisture transport within a clothing system, only the inner layer plays a significant role and that testing the liquid moisture transport capacity of materials used in subsequent layers of clothing is unnecessary. This study demonstrated that adding an additional outer layer, moving from the body outward toward the surrounding air, significantly alters the liquid moisture transport behavior of the inner layer, particularly on the surface adjacent to the skin. Specifically, the performance of the top surface of the next-to-skin layer in terms of liquid moisture transport deteriorates when another outer layer is added. The extent of these changes depends on the weave structure of the fabrics used. Among the tested woven fabrics, the plain weave showed the least deterioration in moisture transport performance in both two- and three-layer assemblies. In contrast, the twill 3/1 S fabric exhibited the greatest changes in moisture transport behavior when layered.

In two-layer assemblies where the outer layer features a different weave structure, the moisture transport properties of the inner layer’s inner surface are influenced by the characteristics of the outer fabric. For the analyzed woven fabrics, it was found that combining a plain weave fabric as the inner (next-to-skin) layer with a twill weave fabric as the outer layer resulted in better moisture transport performance than when both layers were made of plain weave fabric.

These findings underscore the importance of evaluating the moisture transport properties of entire fabric assemblies—not just individual layers—when designing multilayer clothing systems intended to enhance the physiological comfort of the wearer.

## Figures and Tables

**Table 1:** The basic structural parameters of the investigated fabrics.

No.	Weave	Mass per Unit Area g/m^2^	Warp Density -/cm^−1^	Warp Density -/cm^−1^	Thickness mm
1	Plain	240	31.6	11.7	0.61
2	Twill 3/1 S	238	31.7	11.7	0.70
3	Rep 2/2	242	32.0	11.8	0.58

## Data Availability

The original contributions presented in this study are included in the article and Supplementary Materials. Further inquiries can be directed to the corresponding author.

## Supplementary Materials

The following supporting information can be downloaded at: https://www.mdpi.com/article/10.3390/ma18102326/s1, Table S1: Plain woven fabric—1 layer; Table S2: Plain woven fabric—2 layers; Table S3: Plain woven fabric—3 layers; Table S4: Twill woven fabric—1 layer; Table S5: Twill woven fabric—2 layers; Table S6: Twill woven fabric—3 layers; Table S7: Rep woven fabric—1 layer; Table S8: Rep woven fabric—2 layers; Table S9: Rep woven fabric—3 layers; Table S10: The 2-layer set: plain/twill. The results from the MMT M290 are presented in Supplementary Materials in the Tables S1–S10.
