# Supplementary material for: Liquid Moisture Transport in Single and Layered Cotton Woven Fabrics
Source: Materials (Basel). 2025 May 16;18(10):2326. doi: 10.3390/ma18102326 (PMC12112796; doi:10.3390/ma18102326)
Supplement: Supplementary file 1 [file materials-18-02326-s001.zip › materials-3604253-supplementary.pdf]

RESULTS OF FABRICS' MEASUREMENT USING THE MMT M290

Table S1. Plain woven fabric – 1 layer

Specimen	Top	Bottom	Top	Bottom	Top	Bottom	Top	Bottom	Accumulative one-way transport index	OMMC
	Wetting Time	Wetting Time	Absorption Rate	Absorption Rate	Max Wetted Radius	Max Wetted Radius	Spreading Speed	Spreading Speed		
	[s]	[s]	[%/s]	[%/s]	[mm]	[mm]	[mm/sec]	[mm/sec]	[%]	[-]
1	2.808	3.089	70.6871	64.5419	20	25	5.3705	5.6792	-64.1595	0.4015
2	3.276	3.37	73.413	66.0591	25	25	5.9252	5.772	-73.0339	0.4057
3	3.65	3.744	74.1378	68.438	20	25	4.946	5.1889	-19.0924	0.4467
4	3.932	4.025	75.7173	71.3563	25	20	5.3328	4.9267	-7.0275	0.4682
5	4.961	4.493	84.5671	76.6436	25	20	5.1943	4.7571	-53.6416	0.4351

Table S2. Plain woven fabric – 2 layers

Specimen	Top	Bottom	Top	Bottom	Top	Bottom	Top	Bottom	Accumulative one-way transport index	OMMC
	Wetting Time	Wetting Time	Absorption Rate	Absorption Rate	Max Wetted Radius	Max Wetted Radius	Spreading Speed	Spreading Speed		
	[s]	[s]	[%/s]	[%/s]	[mm]	[mm]	[mm/sec]	[mm/sec]	[%]	[-]
1	2.808	3.089	70.6871	64.5419	20	25	5.3705	5.6792	-64.1595	0.4015
2	3.276	3.37	73.413	66.0591	25	25	5.9252	5.772	-73.0339	0.4057
3	3.65	3.744	74.1378	68.438	20	25	4.946	5.1889	-19.0924	0.4467
4	3.932	4.025	75.7173	71.3563	25	20	5.3328	4.9267	-7.0275	0.4682
5	4.961	4.493	84.5671	76.6436	25	20	5.1943	4.7571	-53.6416	0.4351

Table S3. Plain woven fabric – 3 layers

Specimen	Top Wetting Time [s]	Bottom Wetting Time [s]	Top Absorption Rate [%/s]	Bottom Absorption Rate [%/s]	Top Max Wetted Radius [mm]	Bottom Max Wetted Radius [mm]	Top Spreading Speed [mm/sec]	Bottom Spreading Speed [mm/sec]	Accumulative one-way transport index [%]	OMMC [-]
1	4.587	4.961	48.9347	46.7363	15	15	2.4977	2.4092	41.425	0.3211
2	6.177	5.99	48.2437	44.1765	15	15	2.1466	2.0966	43.4266	0.2901
3	5.242	4.774	49.0634	51.8736	20	20	2.6224	3.0373	26.8811	0.3715
4	7.114	5.523	48.8284	50.1497	20	20	2.8558	2.7243	103.1519	0.4254
5	5.429	5.242	48.9507	48.4939	20	15	2.6602	2.4936	83.1696	0.3794

Table S4. Twill woven fabric – 1 layer

Specimen	Top Wetting Time [s]	Bottom Wetting Time [s]	Top Absorption Rate [%/s]	Bottom Absorption Rate [%/s]	Top Max Wetted Radius [mm]	Bottom Max Wetted Radius [mm]	Top Spreading Speed [mm/sec]	Bottom Spreading Speed [mm/sec]	Accumulative one-way transport index [%]	OMMC [-]
1	0.187	0.187	48.1019	38.0962	20	20	20.0764	20.0079	-238.258	0.328
2	3.089	2.995	59.1818	48.3945	20	20	4.4397	4.2226	-205.981	0.3567
3	3.089	3.089	59.625	46.2837	20	20	4.2402	4.0233	-209.038	0.3508
4	3.182	3.089	63.9674	47.715	20	20	4.3928	4.1132	-204.279	0.3548
5	2.715	2.902	62.3716	49.3023	20	20	4.4745	4.2073	-185.893	0.3592

Table S5. Twill woven fabric – 2 layers

Specimen	Top Wetting Time	Bottom Wetting Time	Top Absorption Rate	Bottom Absorption Rate	Top Max Wetted Radius	Bottom Max Wetted Radius	Top Spreading Speed	Bottom Spreading Speed	Accumulative one-way transport index	OMMC
	[s]	[s]	[%/s]	[%/s]	[mm]	[mm]	[mm/sec]	[mm/sec]	[%]	[-]
1	4.025	4.118	41.9	33.8718	15	15	2.7378	2.5672	-102.148	0.1969
2	3.931	4.493	44.0652	34.6591	15	15	2.6641	2.3331	-132.421	0.1796
3	4.212	4.212	45.0245	34.273	15	15	2.5985	2.3625	-146.578	0.181
4	3.089	4.118	44.2079	34.1985	15	15	2.8978	2.3994	-154.864	0.1838
5	3.931	4.025	42.1566	35.3864	15	15	2.5038	2.6234	-74.3435	0.2058

Table S6. Twill woven fabric – 3 layers

Specimen	Top Wetting Time	Bottom Wetting Time	Top Absorption Rate	Bottom Absorption Rate	Top Max Wetted Radius	Bottom Max Wetted Radius	Top Spreading Speed	Bottom Spreading Speed	Accumulative one-way transport index	OMMC
	[s]	[s]	[%/s]	[%/s]	[mm]	[mm]	[mm/sec]	[mm/sec]	[%]	[-]
1	4.025	5.897	32.1406	26.3778	15	10	2.2221	1.498	-120.755	0.087
2	3.651	5.429	34.4126	27.8846	15	10	2.4258	1.8275	-120.756	0.1186
3	3.837	5.241	32.8508	26.0244	10	10	1.8637	1.4607	-99.6193	0.0829
4	5.428	5.896	31.4013	26.6216	15	10	2.1465	1.3901	-81.9104	0.0787
5	4.212	5.616	32.0178	25.9135	15	15	2.1407	1.9413	-79.3233	0.1226

Table S7. Rep woven fabric – 1 layer

Specimen	Top	Bottom	Top	Bottom	Top	Bottom	Top	Bottom	Accumulative one-way transport index	OMMC
	Wetting Time	Wetting Time	Absorption Rate	Absorption Rate	Max Wetted Radius	Max Wetted Radius	Spreading Speed	Spreading Speed		
	[s]	[s]	[%/s]	[%/s]	[mm]	[mm]	[mm/sec]	[mm/sec]	[%]	[-]
1	3.182	3.089	64.5121	58.6385	20	20	4.0795	4.0466	-25.0609	0.4128
2	3.276	3.464	63.5217	59.2819	20	20	4.1258	3.9295	-41.3284	0.3907
3	3.088	3.556	63.6328	58.7066	20	20	4.0733	3.9798	-14.4177	0.4232
4	3.276	3.276	64.8116	58.4339	20	20	4.1537	4.7701	-47.9674	0.3868
5	3.369	3.276	63.5775	56.5722	20	20	4.0553	4.7862	-43.7379	0.3863

Table S8. Rep woven fabric – 2 layers

Specimen	Top	Bottom	Top	Bottom	Top	Bottom	Top	Bottom	Accumulative one-way transport index	OMMC
	Wetting Time	Wetting Time	Absorption Rate	Absorption Rate	Max Wetted Radius	Max Wetted Radius	Spreading Speed	Spreading Speed		
	[s]	[s]	[%/s]	[%/s]	[mm]	[mm]	[mm/sec]	[mm/sec]	[%]	[-]
1	3.838	4.213	45.1049	42.8028	15	15	2.4464	2.3623	29.7647	0.2933
2	3.65	4.118	45.4214	43.582	15	15	2.5682	2.4579	14.1455	0.286
3	3.276	3.838	44.8866	44.7261	15	15	2.7945	2.6986	41.706	0.3399
4	4.586	4.212	43.5169	42.2135	15	15	2.2982	2.3482	29.2424	0.2899
5	3.931	4.399	43.5405	44.031	15	15	2.6476	2.4404	4.4607	0.2751

Table S9. Rep woven fabric – 3 layers

Specimen	Top Wetting Time [s]	Bottom Wetting Time [s]	Top Absorption Rate [%/s]	Bottom Absorption Rate [%/s]	Top Max Wetted Radius [mm]	Bottom Max Wetted Radius [mm]	Top Spreading Speed [mm/sec]	Bottom Spreading Speed [mm/sec]	Accumulative one-way transport index [%]	OMMC [-]
1	4.961	6.365	31.7016	35.4445	15	10	1.8464	1.3566	42.7375	0.2034
2	5.522	4.773	33.991	31.0036	10	10	1.4693	1.6593	-2.3351	0.1662
3	5.148	5.241	30.0811	33.0353	10	10	1.4558	1.4835	85.645	0.255
4	4.68	6.084	33.2011	34.3333	15	10	1.8593	1.4332	30.5975	0.1932
5	4.868	5.71	33.1644	34.4975	15	10	1.999	1.3716	-11.6694	0.1416

Table S10. The 2-layer set: plain/twill

Specimen	Top Wetting Time [s]	Bottom Wetting Time [s]	Top Absorption Rate [%/s]	Bottom Absorption Rate [%/s]	Top Max Wetted Radius [mm]	Bottom Max Wetted Radius [mm]	Top Spreading Speed [mm/sec]	Bottom Spreading Speed [mm/sec]	Accumulative one-way transport index [%]	OMMC [-]
1	5.616	3.651	34.5588	41.1668	15	15	2.1486	3.034	173.8864	0.5048
2	5.709	3.65	35.5001	43.4075	15	20	2.0831	3.5555	171.5506	0.5519
3	5.616	3.37	33.8253	42.6056	10	20	1.4012	3.6513	220.4838	0.6121
4	5.428	3.556	34.4841	41.6598	15	20	2.2042	3.5135	183.5097	0.5569
5	5.709	3.556	35.0472	43.2636	15	20	2.2094	3.4113	222.117	0.5957
